# Effect of Doublesynch and Estradoublesynch protocols on estrus induction, conception rate, plasma progesterone, protein, and cholesterol profile in anestrus Gir heifers

**DOI:** 10.14202/vetworld.2018.542-548

**Published:** 2018-04-27

**Authors:** N. J. Chaudhary, D. M. Patel, A. J. Dhami, K. B. Vala, K. K. Hadiya, J. A. Patel

**Affiliations:** 1 Department of Animal Reproduction, Gynaecology and Obstetrics, College of Veterinary Science and Animal Husbandry, Anand Agricultural University, Anand - 388 001, Gujarat, India; 2 Department of Animal Reproduction, Gynaecology and Obstetrics, College of Veterinary Science and Animal Husbandry, Junagadh Agricultural University, Junagadh - 362 001, Gujarat, India

**Keywords:** cholesterol, conception rate, estrus synchronization, Gir heifers, progesterone, proteins, pubertal anestrus

## Abstract

**Aim:**

This study aimed to evaluate the efficacy of Doublesynch and Estradoublesynch protocols on estrus induction, conception rates, plasma progesterone, protein, and cholesterol profile in anestrus Gir heifers.

**Materials and Methods:**

In this study, 50 pubertal anestrus Gir heifers were selected from the field and farm conditions. The heifers were dewormed (injection ivermectin, 100 mg, s/c) and supplemented with minerals and vitamins (injection organic phosphorus 800 mg and injection Vitamin AD_3_E and Biotin 10 ml i/m) and multi-mineral bolus at 1 bolus daily for 7 days. The heifers were randomly divided into three groups: Doublesynch (n=20), Estradoublesynch (n=20), and control (n=10). The animals were monitored for estrus response, estrus interval, behavioral signs, and conception rates after induced/first, second, and third cycle post-treatment. Blood samples were obtained on day 0, day 9, day 12, and on day 12 post-artificial insemination (AI) for determination of plasma progesterone, protein, and cholesterol profile.

**Results:**

The estrus response rate between Doublesynch and Estradoublesynch protocols was similar between treated heifers (85% and 95%). The interval from the second prostaglandin F2α (PGF_2_α) injection to estrus induction did not differ between the groups (63.87±4.19 vs. 58.27±3.83 h). The conception rates following induced estrus (20% vs. 30%), at the second cycle (23.07% vs. 16.66%), at the third cycle (22.22% vs. 30.00%), and the overall conception rate (45% and 55%) within 27.89±5.75 and 26.45±5.48 days were the same across the treatment groups. The mean plasma progesterone concentrations were significantly (p<0.01) higher on day 9 (second PGF_2_α injection) and day 12 post-AI compared to day 0 (first PGF_2_α injection) and the day of fixed-timed artificial insemination. The concentrations were also significantly (p<0.05) higher in conceived than non-conceived heifers on day 9 of treatment and day 12 post-AI in both the protocols. The mean plasma cholesterol concentrations were significantly higher during peak follicular and luteal phases compared to the initial anestrus phase in both the protocols. The values were also higher in non-conceived than conceived animals in both the protocols. The plasma protein profile was not influenced by the sampling days or conceived and non-conceived status.

**Conclusion:**

The results showed that both Doublesynch and Estradoublesynch protocols resulted in similar estrus induction and conception rates with modulation of plasma progesterone and cholesterol profile in anestrus Gir heifers.

## Introduction

Gir cattle, the famous Indian milch breed native to Gir forest in Gujarat, is the hardiest of high yielders in the world [1]. It is one of the best native milch breeds of zebu cattle adapted to the hot, humid climate and diseases of tropics in India and even abroad. However, they are slow breeders and have extended post-pubertal and postpartum anestrus periods compared to their temperate counterparts [2]. Despite of having attained pubertal age and body weight, a large percentage of Indian zebu heifers fail to commence cyclicity [3].

Delayed onset of puberty necessitates exogenous intervention to induce ovarian activity. Several hormonal preparations and protocols have been used to induce estrus in acyclic cattle [4-7]. Estrus synchronization protocols involve the sequential administration of reproductive hormones to manipulate the estrous cycle to provide a fertile oocyte for insemination at a predictable moment. The new protocols have incorporated strategies to adjust the endocrine milieu and consequently support specific portions of the synchronization [5,8,9]. Further, evaluating plasma progesterone and biochemical constituents of blood has a great value in evaluating the reproductive and physiological statuses of the animal, as these have been reported to affect fertility status of bovines [10]. The progesterone hormone is responsible for stimulation of cyclicity, follicular development, and maintenance of pregnancy. Protein deficiency retards the development of reproductive organs and is considered to be a factor responsible for failure or delay in the onset of postpartum estrus [11]. Cholesterol, the most important sterol, is an essential precursor of steroid hormones in the body.

However, studies on the use of recently developed estrus synchronization protocols, namely Ovsynch, controlled internal drug release (CIDR), Doublesynch, and Estradoublesynch in pubertal anestrus heifers of zebu cattle breeds are meager in the literature [12]. Even whether plasma protein and cholesterol levels are altered by these protocols of estrus synchronization is also scarce in cattle [13]. Moreover, the estrus response and conception rates with the use of Doublesynch and Estadoublesynch protocols in both cyclic and acyclic bovines were quite encouraging in earlier studies [14-17]. Hence, this study was aimed to evaluate if Doublesynch and Estradoublesynch protocols induce successful ovulatory estrus, modulate plasma progesterone, protein, and cholesterol profile, and enhance fertility in pubertal anestrus Gir heifers under field conditions.

## Materials and Methods

### Ethical approval

The prior approval from the Institutional Animal Ethics Committee was obtained for the use of farm animals in this study.

### Selection and pre-synchronization treatment of animals

The study was carried out during August 2016 to May 2017 on 50 pubertal anestrus Gir heifers of Baroda District Milk Union, Vadodara, as well as from villages of Junagadh district in Gujarat. The anestrus heifers selected were in the age group of 30-42 months and having average body condition score (BCS, 2.5-3.5) with smooth, small inactive ovaries that were confirmed by twice rectal palpation 10 days apart. All these animals were initially injected once with 100 mg ivermectin s/c, injection organic phosphorus 800 mg, and multivitamins AD_3_E 10 ml i/m, and were supplemented with multimineral bolus at 1 bolus daily for 7 days only to control parasitism, improve nutritional status, and thereby to improve response to hormone therapy. They were then randomly divided into 2 equal groups of 20 heifers each and were subjected to the following two estrus synchronization protocols, keeping one group of 10 animals as an untreated control.

### Synchronization protocols

Under Doublesynch protocol, the heifers received i/m injection of Cloprostenol sodium 500 µg on day 0, injection buserelin acetate, a GnRH analog 20 µg on day 2, second injection of Cloprostenol sodium 500 µg on day 9 and 10 µg GnRH on day 11, followed by fixed-timed artificial insemination (FTAI) twice at 16 and 24 h later, while in Estradoublesynch protocol, the cows received an injection of estradiol benzoate 1 mg on day 10, in place of the second GnRH injection on day 11 in Doublesynch with FTAI twice at 48 and 60 h post-estradiol injection. Ten anestrus heifers kept without any hormonal intervention and followed for spontaneous estrus served as control. Animals inseminated at induced/spontaneous estrus, if not conceived, were followed for the next two cycles. In non-return cases, pregnancy was confirmed by per-rectal examination 60 days of the last AI. Among non-pregnant animals, the ovarian status was also checked as to whether the animal was cyclic or has turned out to be anestrus again.

### Blood sampling and assay procedure

Blood samples were collected from jugular veins in heparinized vacutainers on day 0 – just before treatment, day 9 – at the time of PGF_2_α administration, day 12 – induced estrus/FTAI, and on day 12 post-AI. The blood samples were centrifuged at 3000 rpm for 15 min. The plasma separated out was stored in a deep freezer at −20°C with a drop of sodium merthiolate (0.1%). The plasma progesterone concentrations were measured by employing standard radioimmunoassay technique of Kubasic *et al*. [18]. Labeled antigen (I^125^), antibody-coated tubes, and standards were procured from Immunotech-SAS, Masrsielle-13009, Cedex, France. The plasma total protein and total cholesterol concentrations were determined by Biuret and CHOD/PAP method, respectively, using standard procedures and assay kits with the help of a chemistry analyzer(Mindray, BS 120, Nanshan, Shenzhen-518057, China).

### Statistical analysis

The data on estrus induction response and conception rates were analyzed using Chi-square test, and those of plasma progesterone, total cholesterol, and total protein profile using ANOVA and Duncan’s multiple range test or “*t-*” test employing Statistical Package for Social Sciences (SPSS, USA) software version 20.00 to know the variations between sampling days, treatment groups, and conceived/non-conceived status [19].

## Results and Discussion

### Estrus induction response and conception rates

The behavioral estrus induced in pubertal anestrus Gir heifers under Doublesynch and Estradoublesynch protocols (85 vs. 95%) and the mean intervals from the second PGF_2_α injection to estrus (63.87±4.19 vs. 58.27±3.83 h) did not differ significantly. The intensity of signs of induced estrus under Doublesynch and Estradoublesynch protocols was prominent in 50% and 65%, moderate in 25% and 25%, and weak in 10% and 5% heifers, respectively, while 15% and 5% animals under respective protocols did not show any behavioral estrus signs. Among the control group, only two animals (20%) exhibited spontaneous estrus during the period of 90-day follow-up (Table-1). The estrus synchronization rates achieved with Doublesynch and Estradoublesynch protocols concurred well with previous reports in crossbred anestrus cattle [13] and in acyclic buffaloes [14,20], but were higher than 70% each reported by Parida *et al*. [21] in buffaloes. Moreover, the mean estrus induction intervals recorded with both the protocols corroborated well with earlier reports on anestrus cattle [13] and buffaloes [20,21].

The conception rates in heifers at induced/first, second, third cycle, and overall of 3 cycles following Doublesynch (20.00%, 23.07%, 22.22%, and 45.00%, respectively) and Estradoublesynch protocols (30.00%, 16.66%, 30.00%, and 55.00%, respectively) were statistically same across the treatments (Table-1). Moreover, among the 11 and 9 non-conceived Gir heifers under Doublesynch and Estradoublesynch protocols, 7 and 6 remained cyclic, while 4 and 3 turned out to be anestrus by 60 days of estrus induction/FTAI as revealed by ovarian status. Overall 50% success rate of fertility was achieved in just average 27 days from the start of two treatment protocols, which was much higher than only 10% found in control group.

**Table-1:** Effect of Doublesynch and Estradoublesynch protocols on estrus induction response, estrus induction intervals, and conception rates in pubertal anestrus Gir heifers.

Treatment groups	No.	Estrus induction response (%)	PGF_2_a injection to estrus induction interval (h)	Conception rate (%)				Status of NP Gir heifers at 60-day post-AI		Initiation of treatment to conception (days)

				Induced/first estrus	Second cycle	Third cycle	Overall of 3 cycles	Cyclic	Anestrus	
Doublesynch	20	85.00 (n=17)	63.87±4.19	20.00 (4/20)	23.07 (3/13)	22.22 (2/9)	45.00 (9/20)	7	4	27.89±5.75
Estradoublesych	20	95.00 (n=19)	58.27±3.83	30.00 (6/20)	16.66 (2/12)	30.00 (3/10)	55.00 (11/20)	6	3	26.45±5.48
Anestrus control	10	20.00 (n=2)	-	50.00 (1/2)	0.00 (0/1)	00.00 (0/1)	10.00 (1/10)	1	8	83.00*

NP=Nonpregnant, PGF_2_a: Prostaglandin F2a.

* From the initiation of the experiment. Statistically, the values were similar between protocols

The conception rates obtained at induced estrus with Doublesynch and Estradoublesynch protocols were in accordance with the reports of Roodbari *et al*. [16] as 18.7% and 26.2% in Holstein Friesian cows and with the reports of Prajapati *et al*. [13] as 40% and 30% in crossbred cows. However, other researchers found much higher conception rates of 43.00% and 59.45% at induced estrus in anestrus cattle following the use of Doublesynch [17] and Estradoublesynch [22] protocols, respectively. Further, the present conception rate at FTAI under Doublesynch protocol is comparable to 28.57% recorded in cattle [15], but it is much lower than 55-58% reported in acyclic buffaloes [20,23].

The present overall conception rate of 45% with Doublesynch protocol is also much lower than 80.00%, 72.80% and 71.43% obtained in anestrus cattle by Prajapati *et al*. [13],Abubaker *et al*. [15], and Ozturk *et al*. [17], respectively. The present overall conception rate of 55% with Estradoublesynch protocol is, however, comparable with 55% and 60% obtained with Doublesynch and Estradoublesynch protocols in an earlier study in cattle [24]. In two more studies, the conception rate with Estadoublesynch protocol was found to be 60%, and 64% in anestrus crossbred cattle and buffaloes, respectively [13-14], Our results for the first service and overall 3 cycles’ conception rates with Doublesynch and Estadoublesynch protocols were also comparable with those of Patel *et al*. [25] in anestrus buffaloes from middle Gujarat. The present relatively lower and same conception rates found across treatments could be attributed to identical estrus response, the subject being heifers of just average BCS with small cervix, frightening and struggle while catching for AI from loose housing paddock, and AI being performed by semi-skilled inseminators under field conditions.

### Plasma progesterone profile

In bovines, corpus luteum (CL) or luteinized follicle is the principal source of progesterone hormone *in vivo*, and it is responsible for the stimulation of cyclicity, follicular development, and maintenance of pregnancy. Its estimation in blood or milk reflects the ovarian response to gonadotropins and/or prostaglandins and thereby ovarian dynamics or pregnancy. The overall mean plasma progesterone concentrations were low or basal on the day of initiation of treatment in both the protocols. This suggested the true anestrus status of Gir heifers selected for the study. Further, the mean concentrations on day 9 of treatment, i.e., just before the second PGF_2_α injection, were found to be significantly higher than on day 0 under Doublesynch (1.23±0.12 ng/ml) and Estradoublesynch (1.40±0.20 ng/ml) protocols. This might be due to luteinization of some of the growing follicles and/or ovulation of dominant follicle and formation of CL under the influence of the first GnRH injection. Thereafter, within 2-3 days of the second PGF_2_α injection, the concentrations dropped significantly to the basal levels with induced estrus, when FTAIs were done. These levels again increased significantly (p<0.05) on day 12 post-AI in both the groups with mean values of 2.41±0.38 and 2.55±0.41 ng/ml, respectively. This could be due to ovulatory-induced estruses with development and maintenance of CL and establishment of pregnancy in varying number of animals in each group (Table-2). Further, in Estradoublesynch protocol, the estradiol benzoate injected on day 10 might have triggered positive feedback effect on hypothalamus and pituitary glands resulting in ovulatory luteinizing hormone surge and thereby improved conception rate in that group. The present trend of plasma progesterone compared well with the earlier reports on cattle [26-27] and buffaloes [25].

**Table-2:** Plasma progesterone, total protein, and total cholesterol concentrations in pubertal anestrus Gir heifers on different days of various estrus induction/synchronization protocols and day 12 post-AI.

Plasma profile	Synchronization protocol	Status	No. of heifers	Days from treatment/AI				

				D0	D9	D12, FTAI	D12 post-AI	Overall
Progesterone (ng/ml)	Doublesynch	Conceived	4	0.72±0.08	1.80±0.31^q^	0.48±0.02	5.30±0.56^q^	2.08±0.52^q^
		Non-concd	16	0.53±0.06	1.09±0.11^p^	0.56±0.05	1.69±0.21^p^	0.96±0.08^p^
		Overall	20	0.56±0.05^x^	1.23±0.12^y^	0.54±0.04^x^	2.41±0.38^z^	1.19±0.13
	Estradoublesynch	Conceived	6	0.44±0.04	2.46±0.40^q^	0.53±0.05	5.02±0.49^q^	2.11±0.42^q^
		Non-concd	14	0.58±0.05	0.95±0.07^p^	0.54±0.05	1.49±0.17^p^	0.89±0.07^p^
		Overall	20	0.54±0.04^x^	1.40±0.20^y^	0.53±0.04^x^	2.55±0.41^z^	1.25±0.15
Protein (g/dl)	Doublesynch	Conceived	4	7.20±0.09	7.27±0.10	7.32±0.12	7.25±0.12	7.26±0.05
		Non-concd	16	7.02±0.24	7.27±0.27	7.28±0.22	7.25±0.22	7.21±0.12
		Overall	20	7.06±0.19	7.27±0.21	7.29±0.18^a^	7.26±0.18^a^	7.22±0.09^a^
	Estradoublesynch	Conceived	6	7.41±0.26	7.47±0.25	7.85±0.20	8.09±0.28	7.70±0.13
		Non-concd	14	7.17±0.20	7.44±0.18	7.72±0.17	7.76±0.16	7.52±0.09
		Overall	20	7.24±0.16^x^	7.45±0.14^×y^	7.76±0.13b^y^	7.86±0.14^by^	7.58±0.07^b^
Cholesterol (mg/dl)	Doublesynch	Conceived	4	117.88±8.56	120.10±7.40	137.6850±6.08	142.98±10.31	129.67±4.63^p^
		Non-concd	16	127.45±3.68	134.83±3.33	145.8113±4.23	149.97±3.92	139.51±2.17^q^
		Overall	20	125.53±3.41^x^	131.88±3.25^ax^	144.19±3.61^y^	148.58±3.68^y^	137.54±2.00^a^
	Estradoublesynch	Conceived	6	120.88±4.41^p^	131.75±3.79^p^	143.79±2.34^p^	137.41±1.24^p^	133.46±2.30^p^
		Non-concd	14	134.40±3.17^q^	147.47±3.54^q^	158.84±4.57^q^	160.88±4.91^q^	150.39±2.45^q^
		Overall	20	130.34±2.89^x^	142.76±3.14^by^	154.32±3.60^z^	153.83±4.21^z^	145.32±2.03^b^

D-0=Day of starting the treatment, D-9=Day of PG injection, While D-12=Fixedtimed artificial insemination, Non-concd=Non-conceived. Means of a trait bearing uncomon superscripts within the row (x, y, z), column (a, b), and conceived and nonconceived (p, q) subgroups within the protocol differ significantly (p<0.05). FTAI=Fixed-timed artificial insemination

The mean plasma progesterone concentrations were higher (p<0.05) in conceived than non-conceived cows on day 12 post-AI in both Doublesynch and Estradoublesynch protocols with values of 5.30±0.56 versus 1.69±0.21 and 5.02±0.49 versus 1.49±0.17 ng/ml, respectively. Further, significant differences were also found in conceived and non-conceived animals on day 9 of treatment (Table-2), indicating better luteal activity before induced estrus in conceived animals. Significantly lower plasma progesterone profile seen on day 12 post-AI in non-conceived cows under both the protocols proved that these were the cases of anovulatory estrus and/or luteal insufficiency. Earlier workers [12,28-29] observed significantly (P<0.05) higher plasma progesteronevalues on day 20-21 post-AI in conceived than non-conceived anestrus zebu cattle with Ovsynch and CIDR protocols. Oyedipse *et al*. [30] observed nonsignificantly higher plasma progesterone values for the pregnant than non-pregnant heifers following synchronization of estrus and AI. The trend and mean plasma progesterone concentrations found in the present study for the effect of sampling days and conceived and nonconceived statuses of animals closely collaborated with previous reports [4,25,29,31] following use of various estrus induction/synchronization protocols in anestrus cattle and buffaloes.

### Plasma total protein profile

In heifers under Doublesynch protocol, the overall mean plasma protein concentrations did not differ significantly between sampling days. A similar non-significant variation in mean plasma protein levels in anestrus Gir [28] and crossbred [32] cattle treated with Ovsynch and CIDR protocols have been reported earlier. In Estradoublesynch protocol, the mean plasma protein levels on the day of FTAI and day 12 post-AI were significantly (p<0.05) higher compared to day 0 and 9 of the protocol. Recent studies [13,33-34] also reported similar findings with different synchronization protocols in postpartum anestrus crossbred cows. Further, the overall mean protein concentrations did not differ significantly between the two protocols at day 0 and day 9. However, heifers under Estradoublesynch protocol had significantly (p<0.05) higher values on the day of FTAI and day 12 post-AI as compared to Doublesynch. Similar findings were reported by Ammu *et al*. [28] in Ovsynch- and CIDR-treated anestrus Gir cows and by Patel *et al*. [32] in crossbred cows. The overall pooled mean plasma total protein concentration was significantly lower in anestrus heifers under Doublesynch than Estradoublesynch protocol (7.22±0.09 vs. 7.58±0.07 g/dl). However, in a recent study [24], an inverse trend was observed with higher value in crossbred cattle under Doublesynch protocol than Estradoublesynch protocol (8.19±0.27 vs. 7.31±0.69 g/dl).

Further, the protein profile did not differ significantly between conceived and non-conceived groups at any of the days in any of the protocols. These observations were in close agreement with those in anestrus crossbred cattle with the same protocols [13], and in normal cows [35] that conceived than those did not conceive (6.86±0.10 vs. 6.04±0.10 g/dl). Plasma protein levels change with different stages of reproduction, depending on the feed intake of the animal. Protein deficiency retarded the development of reproductive organs and was considered to be a factor responsible for failure or delay in the onset of postpartum estrus [11]. However, Gentile *et al*. [36] reported that serum protein level was not related to fertility in dairy cows.

### Plasma total cholesterol profile

Significant differences were observed in the overall mean plasma cholesterol concentrations between sampling days in heifers under Doublesynch protocol. The values on day 12 post-AI and day of FTAI were significantly higher as compared to those of day 0 and day 9. Similar results were also found in Estradoublesynch protocol with significantly higher value on day 9 compared to day 0. The mean plasma cholesterol concentration was lowest at initial anestrus phase (day 0), but reached a peak on the day of induced estrus/FTAI (follicular phase) and day 12 post-AI (luteal phase) in both the protocols (Table-2). Bora *et al*. [33] observed higher plasma cholesterol concentrations on the day of induced estrus and on day 20 than day 10 or 0 of treated respondent postpartum anestrus crossbred cows. However, in earlier studies employing Ovsynch, Cosynch, PRID, etc., the plasma cholesterol levels were not influenced significantly between sampling days in anestrus cattle [4,32,34].

There were no significant differences in protein profile between conceived and non-conceived heifers at any of the sampling days, although the values were apparently higher in non-conceived than conceived animals with significant difference in pooled values (139.51±2.17 vs. 129.67 ±4.63 mg/dl) in heifers under Doublesynch protocol, while in heifers under Estradoublesynch protocol, the concentrations were significantly (p<0.01) higher in non-conceived than conceived animals at all days including overall pooled mean (Table-2). Similar findings were also noted earlier [13] with both these protocols in anestrus cows. The increase in circulatory cholesterol level at estrus induction was opined to be due to the mechanism by which estrogens affect the complex interrelationships of pituitary–thyroid–adrenal functions and the estrogens had an effect on the carbohydrate metabolism that in turn caused an increased production of cholesterol in endocrine gland tissue from acetate [37]. Steroid hormones have a direct relationship with cholesterol metabolism. The higher cholesterol level in the cycling animals is indicative of more secretion of steroids during estrus induction due to increased ovarian activity [38]. Kavani *et al*. [39] opined that the low cholesterol level might have resulted in inadequate synthesis of sex steroid hormones leading to anestrus condition.

## Conclusion

From the study, it can be concluded that both Doublesynch and Estradoublesynch protocols resulted in almost similar estrus induction and conception rates, with modulation of plasma progesterone and cholesterol profile. It is, therefore, suggested to use Estradoublesynch protocol due to its cost-effectiveness in the successful treatment of anestrus in pubertal Gir heifers.

## Authors’ Contributions

DMP and AJD planned and designed the study. The experiment was conducted by NJC, KBV, KKH, and JAP. NJC did laboratory work. AJD and DMP carried out interpretation of results. All authors participated in data analysis, preparation of a draft of the manuscript, and read and approved the same. All authors read and approved the final manuscript.
